# Ikelos‐Rating Scale: Validation of a Behavioural Severity Marker in REM Sleep Behaviour Disorder

**DOI:** 10.1111/jsr.70019

**Published:** 2025-02-17

**Authors:** Sophia Stotz, Frederik Bes, Dieter Kunz

**Affiliations:** ^1^ Clinic for Sleep & Chronomedicine St. Hedwig‐Hospital Berlin Germany; ^2^ Sleep Research & Clinical Chronobiology Institute of Physiology, Charité Universitätsmedizin Berlin Germany

**Keywords:** biomarker, DaT‐SPECT, iRBD, RWA, severity scale, videometry

## Abstract

The Ikelos‐Rating Scale (Ikelos‐RS) is a new, expert‐interviewed and bedpartner‐reported severity marker assessing frequency and expression of isolated REM sleep behaviour disorder (iRBD), a precursor of clinical α‐synucleinopathies. This study aimed to validate the Ikelos‐RS in 180 patients with three‐night PSG‐confirmed iRBD (68.4 ± 8.3 years; 139 m). Inter‐rater (*n* = 45) and test–retest reliabilities (*n* = 25; 174 Ikelos‐RS) were evaluated. For construct validity, correlation analyses were performed with: (1) Clinical Global Impressions‐Severity (CGI‐S; *n* = 151), (2) REM sleep without atonia (RWA) within videometry (*n* = 20), (3) RWA at initial diagnosis (*n* = 131) and changes over time (*n* = 36), (4) dopamine transporter scintigraphy (DaT‐SPECT) at baseline and changes over time (*n* = 75). RWA‐ and DaT‐SPECT‐analyses were conducted for the whole sample (‘all’) and after excluding confounders (‘cleaned’). Correlation analyses indicated high inter‐rater (*r*_*s*_ = 0.865, *p* < 0.001) and test–retest reliabilities (*r*_*s*_ = 0.900, *p* < 0.001). Construct validity was supported by associations of Ikelos‐RS with (1) CGI‐S (*r*_*s*_ = 0.845, *p* < 0.001), (2) RWA within videometric analysis (*r*_*s*_ = 0.592, *p* = 0.006) and at baseline (‘all’: *r*_*s*_ = 0.274, *p* = 0.002), (3) DaT‐binding (*z*‐scores) at baseline in right anterior putamen (AP) (‘all’: *r*_*s*_ = −0.319, *p* = 0.005) and changes over time, most pronounced in right anterior putamen (AP) (‘all’: *r*_*s*_ = −0.243, *p* = 0.035; ‘cleaned’: *r*_*s*_ = −0.374, *p* = 0.008) and left posterior putamen (PP) (‘all’: *r*_*s*_ = −0.259, *p* = 0.025; ‘cleaned’: *r*_*s*_ = −0.319, *p* = 0.024). Given its high reliability and construct validity, demonstrated by associations with the best available severity markers DaT‐binding ratios and RWA, Ikelos‐RS appears to represent a reliable, valid and easy‐to‐use tool for measuring the severity of iRBD. Thus, Ikelos‐RS may prove beneficial in research. Its suitability as a screening tool in older at‐risk populations needs to be proven in future studies.

## 1 Introduction

Neurodegenerative diseases, causing immense physical and psychological stress for patients, family caregivers and healthcare systems, are not curable today. However, research on preclinical phases, for example, isolated REM sleep behaviour disorder (iRBD) as a prodromal state of clinical α‐synucleinopathies such as Parkinson's disease and Lewy body dementia, offers new perspectives (Bassetti and Bargiotas 2018; Cesari et al. 2021; Kunz et al. 2021; Schenck et al. 2013). Early biomarker identification has shifted focus from symptom management to disease modification in neurodegeneration (Kunz et al. 2023; Miglis et al. 2021; Postuma et al. 2019).

In iRBD, REM sleep without atonia (RWA) is named ‘neurophysiologic hallmark of RBD’ and recognised as one of the most relevant biomarkers alongside reduced dopamine transporter density (American Academy of Sleep Medicine 2014; Lapierre and Montplaisir 1992; Miglis et al. 2021; Nepozitek et al. 2019; Postuma 2019). Videometric analysis (Cesari et al. 2022; Frauscher et al. 2007; Sixel‐Döring et al. 2011), providing more differentiated information about movement patterns concomitant with RWA, has received less attention but was also identified as an independent biomarker, being related to reduced dopamine transporter density as well (Nepozitek et al. 2021). Consequently, regular symptom monitoring would be suitable for evaluating therapy effectiveness including disease‐modifying treatments. On the other hand, determining biomarkers like RWA and DaT‐density is time‐consuming and previous severity scales for RBD are predominantly based on polysomnography (PSG) (Frauscher et al. 2007; Sixel‐Döring et al. 2011). For this reason, the Ikelos‐Rating Scale (Ikelos‐RS), named after the god of nightmares in Greek mythology, has been developed as a user‐friendly tool for assessing RBD severity in clinical practice, incorporating information from patients' bedpartners. The Ikelos‐RS has been partly validated in a large observational study (Kunz et al. 2021), indicating both acute and long‐term effects of melatonin treatment. The present study evaluates the Ikelos‐RS within an extensive validation analysis. The aim was to supply an easy‐to‐use and publicly available tool for severity in RBD.

## 2 Methods

A total of 400 patients with clinical symptoms of acting‐out dreams underwent video‐PSG between 2004 and 2022 in the Clinic for Sleep & Chronomedicine at St. Hedwig‐Hospital (Berlin) during three consecutive diagnostic nights. Of these, 65 were considered non‐RBD, 31 as secondary RBD (because of obstructive sleep apnea or psychotropics), 49 as converted to α‐synucleinopathy at baseline and 255 as iRBD (according to ICSD‐3 criteria; American Academy of Sleep Medicine 2014). Due to the multi‐stage validation processes conducted over an extended period and the evolving patient population, only 180 patients with iRBD were considered for the final analyses. This reduction was primarily driven by the need to ensure complete datasets for all necessary statistical operations, which enabled a more robust analysis and ensured comparability of results. Within this cohort, all patients received melatonin therapy within a chronobiotic protocol (Kunz et al. 2021). The Ikelos‐RS was evaluated by clinicians (specialists in psychiatry and somnology) at the time of diagnosis and at least once during the course of treatment. Retrospective evaluations were based on medical reports. Reliability was tested between independent raters and two measuring points. Construct validity was evaluated by comparing the results of the Ikelos‐RS with established methods for determining RBD severity, including videometric analyses during PSG, dopamine transporter scintigraphy (DaT‐SPECT), RWA (both with follow‐ups) and the Clinical Global Impressions—Severity Scale (CGI‐S; Guy 2017). These methods were chosen as reference standards due to their combined ability to capture different aspects of RBD severity, providing a comprehensive assessment of the construct validity of the Ikelos‐RS.

For RWA analyses, only data from Night 2 were used, being finally included if at least 30 min of REM sleep could be analysed and an Ikelos‐RS was available within a period of 6 months around PSG (the latter criterion also applies for DaT‐SPECT). For correlation analyses of Ikelos‐RS with RWA and DaT‐SPECT at initial diagnosis and over time, calculations were made for all available data (‘all’) and additionally after exclusion of patients with confounding factors influencing RWA or DaT‐SPECT (‘cleaned’), such as psychotropic medication, beta‐blockers, stimulants, or invasive medical treatments (Chahid et al. 2023; Feemster et al. 2020; Kang and Bega 2024). A complete list of exclusion criteria is provided in the results section (‘*Initial RWA and DaT‐SPECT*’). Criteria for the sample of 20 patients within the videometric analysis were: (1) No confounding medication (e.g., antidepressants), (2) no simultaneous periodic leg movements in all three nights (periodic limb movement index (PLMI) < 15/h), (3) no concurrent respiratory events of obstructive breathing disorders (respiratory disturbance index (RDI) < 15/h) in all three nights, (4) RWA analysis available for at least 2/3 of the time in REM in the polysomnographic nights, (5) comparable RWA between the 3 polysomnographic nights (≤ 1 standard deviation from averaged three nights) and (6) a satisfying videographic quality (i.e., high sharpness, contrast and time‐resolution).

All patients had provided written informed consent for their clinical data to be analysed and published anonymously. The ethics committee of Charité—Universitätsmedizin Berlin approved the publication of the results of the post hoc data analysis.

### 2.1 Polysomnography

Parameters recorded as part of video‐PSG were: EEG (electroencephalography, according to the 10–20 system: F3, F4, C1, C2, O1, O2, A1 and A2), electromyogram (EMG; from chin and mm. tibiales, and, since early 2021, bilateral flexor digitorum superficialis), electrocardiogram (ECG), electrooculogram (EOG), nasal respiratory flow, respiratory effort of thorax and abdomen, core body temperature, oxygen saturation, video (infrared, 25 frames per second) and audio (44.1 kHz, 16 bits, mono). Recordings were made with digital recording systems (Monet 24‐CPU, TMS‐international; Enschede, the Netherlands; Embla N7000, Natus Medical Inc.; Middleton, Wisconsin, USA), using REMbrandt DataLab 7.5 software (Medcare Automation; Amsterdam, the Netherlands); video‐PSG was analysed using REMbrandt Analysis Manager 9.1.5. RWA was calculated with an automatic scoring algorithm based on the chin‐EMG, resulting in output according to the ‘Montréal’‐method (Lapierre and Montplaisir 1992), in addition to a recently developed, concise RWA scoring method (Ikelos‐RWA; Papakonstantinou et al. 2024). In this study, RWA scores generated by Ikelos‐RWA were used for further analyses.

### 2.2 DaT‐SPECT

For DaT‐SPECT, patients were administered 130–185 MBq [^123^I] ioflupane (FPCIT, Datscan, GE Healthcare) intravenously after oral administration of 1000 mg sodium perchlorate to block the thyroid gland. SPECT images were taken with a gamma camera (NM/CT 670, GE Healthcare) approximately 4 h after injection. Data were iteratively reconstructed with HERMES HybridRecon (Koch et al. 2011) and automatically analysed with BRASS ENCDAT (EARL). Output variables were specific binding ratios (SBRs), using ioflupane uptake in the occipital lobe as the background reference region, and *z*‐scores (from comparison with a normal collective of 103 SPECT examinations from the European multicenter database of healthy controls ENC‐DAT) (Varrone et al. 2013) of a total of six regions in the striatum (caudate nucleus [CN], AP, PP—right and left respectively). No adverse events were reported as a result of DaT‐SPECT.

### 2.3 Ikelos‐Rating Scale

The Ikelos‐RS (Kunz et al. 2021) is based on interviews with patients' bedpartners only. Observational periods are the last 6 months or since the last medical consultation if less than 6 months. The Ikelos‐RS consists of two dimensions: On the first scale ‘frequency’ the occurrence of symptoms is estimated from never (=0) to less than 2–3 times per month (=1), 1–2 times per week (=2), 3–5 times per week (=3) or daily (=4), subsuming all motor events and vocalisations. On the ‘expression’ scale, the most severe manifestation of motor events is recorded (talking or slight distal movements = 0, shouting, laughing or complex movements without aggressive connotation = 1, complex movements with (risk of) injury = 2, or leaving the bed = 3). All RBD‐relevant particularities (e.g., influencing co‐medication like antidepressants) are noted. Simultaneously to the Ikelos‐RS, two components of the CGI (CGI‐S and CGI Improvement Scale (CGI‐I)) were evaluated, both ranging from 0 to 7.

### 2.4 Videometric Analysis

For videometric analysis, all video recordings corresponding to the total REM sleep period were examined. REM episodes were divided into 30‐s epochs. Within each epoch, all motor activity and vocalisations were tabulated. If these were detected, the following information was documented: (1) Anatomical localisation, (2) epoch number and (3) rated value of the Ikelos‐RS scale ‘expression’ Longer movements that extended beyond a single epoch were treated as a single unit if they were part of a continuous and uninterrupted sequence of motor activity. The most severe manifestation in all REM epochs was considered for the Ikelos‐RS total score. While a wide range of movement types during REM sleep were analysed, comfort movements, limb‐like movements (such as brief twitches), arousal‐related movements, and those associated with respiratory events were not included in the analysis. To determine values for the dimension ‘frequency’ the proportion of REM epochs with detected activity in the total number of REM epochs in night 2 was calculated and then set ordinal (0%–20% = 0, 20%–40% = 1, 40%–60% = 2, 60%–80% = 3, 80%–100% = 4), allowing an Ikelos‐RS total score to be generated.

### 2.5 Statistical Analyses

Mean values, standard deviations and ranges were calculated to describe demographic data. Spearman's rank correlations were used to assess test–retest and inter‐rater reliabilities of the Ikelos‐RS and to evaluate its association with other variables at initial and follow‐up examinations, if available. Change scores were generated for repeated Ikelos‐RS, RWA and DaT‐SPECT by calculating differences in parameters between initial and last follow‐up examinations, that is, subtracting the values of the follow‐up from the initial examination. In accordance with the pre‐defined exclusion criteria, data points with non‐evaluable results or missing data were not considered in the analyses. Statistical operations were performed with IBM SPSS Version 28.0 (IBM Corp. Released 2021. IBM SPSS Statistics for Windows, Version 28.0. Armonk, NY: IBM Corp). Required sample sizes were determined a priori using G*Power software (with an effect size of *d* = 0.70, a significance level of *α* = 0.05, and a power of 80%).

## 3 Results

**FIGURE 1:**
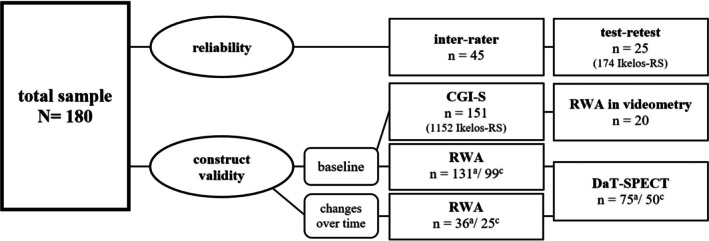
Sample sizes with number of Ikelos‐RS and further examinations considered for individual validation aspects (^a^ = ‘all’; ^c^ = ‘cleaned’).

A total sample of 180 patients, with an average age of 68.40 ± 8.30 years at iRBD diagnosis (139 males), was included in the study. The mean duration between symptom onset and diagnosis was 5.06 ± 4.75 years. Figure 1 illustrates the individual validation steps.

### 3.1 Reliability

Results of Spearman's rank correlation indicated large effects for inter‐rater (*r*_*s*_ = 0.865, *p* < 0.001) and test–retest reliability (*r*_*s*_ = 0.900, *p* < 0.001).

### 3.2 Construct Validity

#### 3.2.1 CGI‐S

Correlation analysis showed a high positive association (*r*_*s*_ = 0.845, *p* < 0.001) between Ikelos‐RS and CGI‐S (Ikelos‐RS score: Mean = 2.55 ± 1.93, range = 0–7.00; CGI‐S: Mean = 2.86 ± 1.50, range = 0–7.00).

#### 3.2.2 RWA in Videometric Analysis

Patients within video analysis (age at iRBD diagnosis: 66.38 ± 9.29 years, 13 male) showed an average total sleep time (TST) of 393.15 ± 65.99 min (range = 247.00–519.00 min) with 83.60 ± 23.27 min (range = 53.00–126.00 min) of REM sleep. In total, videometry of 3188 REM sleep epochs was visually analysed, of which motor acting out and/or vocalisation could be observed in 1252 (39.3%) epochs. Involvement of arms (50%) and legs (18.66%) was recorded most frequently, with only 5% of the events containing vocalisation (e.g., shouting or singing). Table 1 shows descriptive values for both dimensions (‘frequency’ and ‘expression’) and the total score of the Ikelos‐RS within videometric analysis.

**TABLE 1:** Ratings (*n*/%) within videometric analysis (*n* = 20) for Ikelos‐RS total score and both dimensions ‘frequency’ and ‘expression’ (most severe manifestation).

Total score	*n* (%)		‘Frequency’ videometry (%)	‘Frequency’ standard	*n* (%)		‘Expression’ most severe manifestation	*n* (%)	
1	4	(20)	0–20	0 = never	5	(25)	0 = talking/slight distal movements	0	(0)
2	6	(30)	20–40	1 = < 2–3 times/month	5	(25)	1 = shouting/complex movements without aggression	10	(50)
3	1	(5)	40–60	2 = 1–2 times/week	4	(20)	2 = complex movements with (risk of) injury	10	(50)
4	3	(15)	60–80	3 = 3–5 times/week	4	(20)	3 = leaving the bed	0	(0)
5	4	(20)	80–100	4 = daily	2	(10)			
6	2	(10)							
7	0	(0)							

**FIGURE 2:**
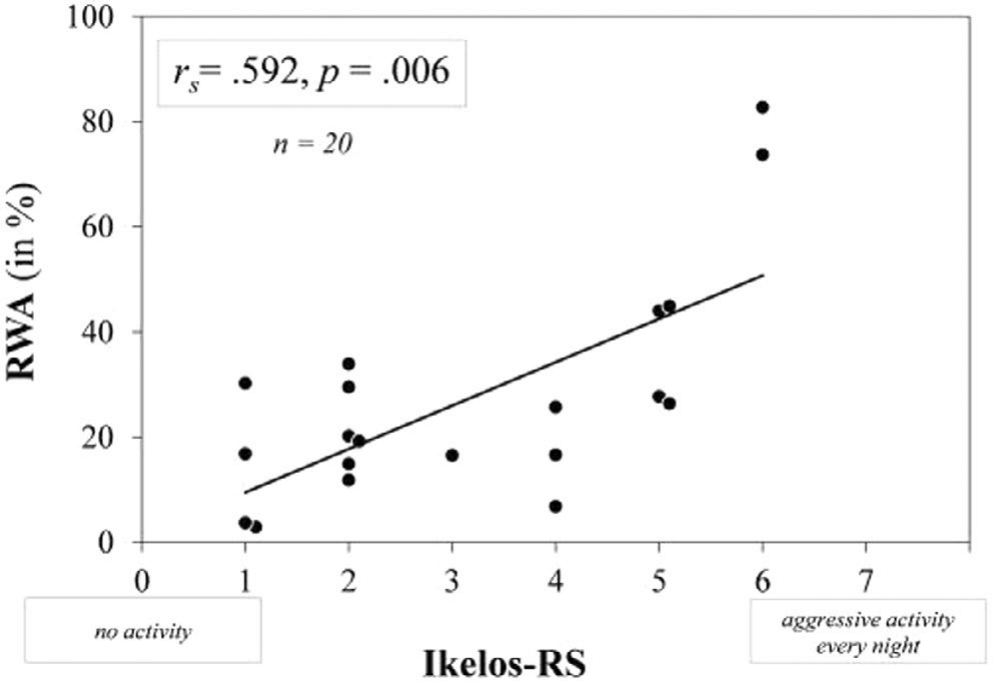
Spearman's rank correlation between Ikelos‐RS total score generated within videometric analysis and RWA (in %) of the same PSG‐night.

Spearman's rank correlation analysis showed a strong association between the Ikelos‐RS total score (mean = 3.15 ± 1.76, range = 1.00–6.00) determined within videometry and RWA (mean = 7.47 ± 20.89, range = 2.90–82.70) of the same night (*r*_*s*_ = 0.592, *p* = 0.006; Figure 2).

#### 3.2.3 Initial RWA and DaT‐SPECT

Data from 131 patients were used for correlation analysis between Ikelos‐RS and RWA at initial PSG (‘all’). Of these, 32 patients were excluded due to antidepressants (*n* = 12), clonazepam (*n* = 2), dopaminergics (*n* = 2), neuroleptics (*n* = 2), opioids (*n* = 1), stimulants (*n* = 1), alcohol abuse (*n* = 4), polypharmacy (*n* = 3), chemotherapy (*n* = 2), testosterone (*n* = 2) and vagus nerve resection (*n* = 1), with 99 patients remaining in the ‘cleaned’ sample.

Spearman's correlation analysis showed small associations between RWA (mean = 35.88 ± 21.56, range = 2.60–95.90) and Ikelos‐RS score (mean = 5.98 ± 1.15, range = 2.00–7.00) at the time of diagnosis (*r*_*s*_ = 0.274, *p* = 0.002) in the total sample (‘all’, 131 patients), but not after exclusion of confounders (‘cleaned’, 99 patients remaining; RWA: Mean = 35.05 ± 20.28, range = 2.60–95.90; Ikelos‐RS score: Mean = 6.00 ± 1.14, range = 2.00–7.00; *r*_*s*_ = 0.129, *p* = 0.202).

Both initial and follow‐up DaT‐SPECTs were undergone by 75 patients (‘all’). Of these, 25 patients were excluded due to factors known to influence Dat‐binding such asantidepressants (*n* = 4), stimulants (*n* = 4), dopaminergics (*n* = 3), neuroleptics (*n* = 2), opioids (*n* = l), polypharmacy (*n* = 3), alcohol abuse (*n* = 2), cortisol (*n* = 2), chemotherapy (*n* = 1), shift work (*n* = 2) and DaT‐SPECT artefacts (*n* = 1). The 50 remaining patients without potential confounders constituted the ‘cleaned’ sample.

Spearman's correlation analyses revealed associations between the Ikelos‐RS (mean = 4.97 ± 1.81, range = 0–7.00) and DaT‐binding in the right AP (SBR: Mean = 2.45 ± 0.52, range = 1.01–3.48; *z*‐score: Mean = −1.49 ± 1.23, range = −5.35 to 1.10) at baseline in the total sample ‘all’ (SBR: *r*_*s*_ = −0.319, *p* = 0.005; *z*‐score: *r*_*s*_ = −0.328, *p* = 0.004), but not after exclusion of confounders in the ‘cleaned’ sample. In contrast, CGI‐S showed no associations with DaT‐binding at baseline in both groups.

#### 3.2.4 Changes Over Time in RWA and DaT‐SPECT

Within 36 patients (‘all’) undergoing repeated PSGs, 11 patients were excluded due to changes in medication such asantidepressants (*n* = 3), dopaminergics (*n* = 2), neuroleptics (*n* = 1), alcohol abuse (*n* = 3), anaesthesia (*n* = 1) and polypharmacy (*n* = 1), with 25 remaining patients representing the ‘cleaned’ sample (follow‐up between first and last PSG: ‘All’ = 3.69 ± 2.68 years, ‘cleaned’ = 3.82 ± 2.66 years).

Change scores between initial and last follow‐up examination were calculated for RWA (mean change score:‘All’ = 4.11 ± 17.72, ‘cleaned’ = 3.03 ± 18.76; range: ‘All’ and ‘cleaned’ = −48.80 to 38.40) and Ikelos‐RS score (mean change score: ‘All’ = −4.00 ± 1.85, ‘cleaned’ = −3.68 ± 1.80; range: ‘All’ = −7.00 to 0, ‘cleaned’ = −6.00 to 0), with negative values indicating clinical improvement over time. Spearman's rank correlation analysis revealed no association between change scores of RWA and Ikelos‐RS score, neither in the total sample (‘all’: *r*_*s*_ = 0.138, *p* = 0.422) nor after excluding potential confounders (‘cleaned’: *r*_*s*_ = 0.161, *p* = 0.442).

For the patients described above with repeated DaT‐SPECT, the follow‐up period between the first and last scan was 3.34 ± 1.94 years in the total sample (‘all’, with 75 patients) and 3.62 ± 1.92 years in the ‘cleaned’ cohort (50 patients). Mean values, standard deviations and ranges for all regions of DaT‐SPECT (SBR and *z*‐scores) and the Ikelos‐RS score of initial and last follow‐up examinations are given in Table 2.

**TABLE 2:** Descriptive statistics of Ikelos‐RS and DaT‐SPECT (SBR and *z*‐score) for the total sample (‘all’; *n* = 75) and after exclusion of confounders (‘cleaned’; *n* = 50) at initial and last follow‐up examinations.

	‘All’ (*n* = 75)				‘Cleaned’ (*n* = 50)			
	Baseline		Last FU		Baseline		Last FU	
	M ± SD	(Min–max)	M ± SD	(Min–max)	M ± SD	(Min–max)	M ± SD	(Min–max)
Ikelos‐RS	4.97 ± 1.81	(0–7.00)	1.77 ± 1.01	(0–6.00)	4.90 ± 1.83	(1.00–7.00)	1.68 ± 0.89	(0–4.00)
DaT‐SPECT (SBR)								
CN right	2.59 ± 0.50	(1.25–3.42)	2.44 ± 0.51	(1.06–3.49)	2.65 ± 0.46	(1.25–3.42)	2.52 ± 0.51	(1.12–3.49)
CN left	2.67 ± 0.48	(1.19–3.62)	2.51 ± 0.49	(1.14–3.59)	2.70 ± 0.44	(1.26–3.44)	2.56 ± 0.50	(1.10–3.59)
AP right	2.45 ± 0.52	(1.01–3.48)	2.30 ± 0.65	(0.58–3.43)	2.50 ± 0.50	(1.01–3.32)	2.37 ± 0.63	(0.80–3.43)
AP left	2.46 ± 0.53	(1.13–3.78)	2.31 ± 0.63	(0.89–3.78)	2.52 ± 0.52	(1.13–3.78)	2.39 ± 0.63	(0.95–3.78)
PP right	1.83 ± 0.55	(0.86–3.14)	1.63 ± 0.61	(0.41–2.79)	1.90 ± 0.53	(0.88–2.93)	1.73 ± 0.56	(0.41–2.79)
PP left	1.90 ± 0.57	(0.58–3.26)	1.70 ± 0.67	(0.56–3.32)	1.97 ± 0.56	(0.58–3.06)	1.80 ± 0.65	(0.77–3.32)
DaT‐SPECT (*z*‐score)								
CN right	−1.46 ± 1.22	(−5.27 to 0.29)	−1.72 ± 1.26	(−5.61 to 0.70)	−1.28 ± 1.14	(−5.27 to 0.29)	−1.48 ± 1.24	(−5.49 to 0.70)
CN left	−1.45 ± 1.20	(−5.43 to 0.78)	−1.72 ± 1.22	(−5.60 to 0.35)	−1.32 ± 1.10	(−5.43 to 0.51)	−1.56 ± 1.25	(−5.74 to 0.35)
AP right	−1.49 ± 1.23	(−5.35 to 1.10)	−1.75 ± 1.56	(−6.23 to 1.16)	−1.33 ± 1.17	(−5.35 to 0.50)	−1.52 ± 1.50	(−5.75 to 1.16)
AP left	−1.52 ± 1.37	(−5.36 to 1.83)	−1.79 ± 1.60	(−5.83 to 2.18)	−1.31 ± 1.34	(−5.36 to 1.83)	−1.53 ± 1.59	(−5.72 to 2.18)
PP right	−2.40 ± 1.36	(−5.00 to 1.01)	−2.78 ± 1.52	(−6.00 to 0.33)	−2.17 ± 1.30	(−5.00 to 0.24)	−2.47 ± 1.39	(−5.74 to 0.33)
PP left	−1.81 ± 1.30	(−4.80 to 1.06)	−2.15 ± 1.53	(−5.15 to 1.19)	−1.61 ± 1.27	(−4.80 to 0.85)	−1.89 ± 1.46	(−4.63 to 1.19)

Abbreviations: ‘all’, total sample; AP, anterior putamen; ‘cleaned’, sample after exclusion of confounders; CN, caudate nucleus; FU, follow‐up; M, mean; Max, maximum; Min, minimum; *n*, number of patients with DaT‐SPECT; PP, posterior putamen; SBR, specific binding ratio; SD, standard deviation.

When calculating change scores between initial and last follow‐up examinations, descriptive analyses showed a small decline in DaT‐binding ratios—comparable to healthy ageing or early RBD (Chahine et al. 2021; Iranzo et al. 2011)—while the overall Ikelos‐RS score improved over time. Associations between change scores in Ikelos‐RS and DaT‐SPECT regions were found in right AP and left PP in both samples. Due to their opposite polarisation, negative correlations indicate that changes in parameters correspond in the same direction (Table 3).

**TABLE 3:** Results for descriptive statistics and Spearman's rank correlation between change scores in Ikelos‐RS and DaT‐SPECT parameters (SBR and *z*‐score) in the total sample (‘all’; *n* = 75) and after exclusion of confounders (‘cleaned’; *n* = 50).

	‘All’ (*n* = 75)					‘Cleaned’ (*n* = 50)				
	Change score		Change per year (%)	*r*_*s*_	*p*	Change score		Change per year (%)	*r*_*s*_	*p*
	M ± SD	(Min–max)				M ± SD	(Min–max)			
Ikelos‐RS	−3.20 ± 2.14	(−6.00 to 2.00)	**—**	**—**	**—**	−3.20 ± 2.27	(−6.00 to 2.00)	**—**	**—**	**—**
DaT‐SPECT (SBR)										
CN right	−0.15 ± 0.49	(−1.61 to 1.41)	−0.08	−0.120	0.305	−0.13 ± 0.47	(−1.09 to 0.95)	−0.66	−0.179	0.213
CN left	−0.15 ± 0.45	(−1.04 to 1.65)	−1.10	−0.173	0.138	−0.13 ± 0.44	(−1.61 to 0.76)	−1.20	−0.225	0.117
AP right	−0.15 ± 0.49	(−1.11 to 0.95)	−1.42	−0.239*	0.039	−0.15 ± 0.37	(−1.04 to 0.56)	−1.43	−0.370**	0.008
AP left	−0.15 ± 0.50	(−1.31 to 1.76)	−1.14	−0.200	0.085	−0.13 ± 0.45	(−1.31 to 0.92)	−0.75	−0.159	0.271
PP right	−0.20 ± 0.41	(−1.29 to 0.87)	−3.07	−0.126	0.282	−0.17 ± 0.38	(−1.29 to 0.63)	−2.41	−0.132	0.360
PP left	−0.19 ± 0.52	(−1.75 to 1.02)	−1.89	−0.253*	0.028	−0.17 ± 0.53	(−1.75 to 1.00)	−0.56	−0.315*	0.026
DaT‐SPECT (*z*‐score)										
CN right	−0.26 ± 1.19	(−3.78 to 3.59)	—	−0.136	0.244	−0.20 ± 1.08	(−3.78 to 2.07)	—	−0.197	0.171
CN left	−0.27 ± 1.10	(−2.34 to 4.20)	—	−0.172	0.141	−0.24 ± 0.91	(−2.34 to 1.46)	—	−0.229	0.109
AP right	−0.26 ± 1.14	(−2.48 to 2.44)	—	−0.243*	0.035	−0.19 ± 1.10	(−2.44 to 2.44)	—	−0.374**	0.008
AP left	−0.28 ± 1.25	(−3.12 to 4.53)	—	−0.203	0.081	−0.22 ± 1.10	(−3.12 to 2.45)	—	−0.169	0.242
PP right	−0.38 ± 1.00	(−3.04 to 2.19)	—	−0.129	0.270	−0.31 ± 0.93	(−3.04 to 1.62)	—	−0.132	0.359
PP left	−0.34 ± 1.16	(−3.74 to 2.45)	—	−0.259*	0.025	−0.28 ± 1.20	(−3.74 to 2.45)	—	−0.319*	0.024

Abbreviations: ‘all’, total sample; AP, anterior putamen; ‘cleaned’, sample after exclusion of confounders; CN, caudate nucleus; M, mean; Max, maximum; Min, minimum; *n*, number of patients with DaT‐SPECT; PP, posterior putamen; *r*_*s*_ , Spearman's rank correlation with Ikelos‐RS change score; SBR, specific binding ratio; SD, standard deviation.

* Significance at the 0.05 level.

** Significance at the 0.01 level.

**FIGURE 3:**
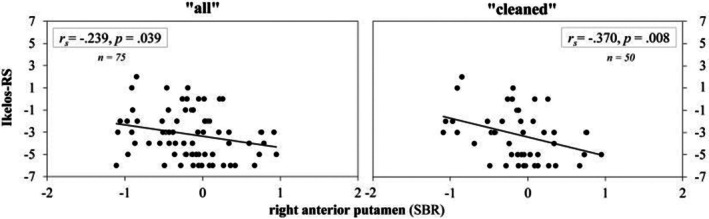
Spearman's rank correlation between change scores of the right anterior putamen (SBR) and Ikelos‐RS score in the total sample (left, ‘all’: 75 patients) and after exclusion of confounders (right, ‘cleaned’: 50 patients). For Ikelos‐RS, positive values indicate an increase (=deterioration), negative values a reduction (=improvement) in symptom severity over time. Interpretation of DaT‐SPECT is reversed: Positive values indicate an increase—which is unusual in healthy subjects and more so in patients with α‐synucleinopathies—whereas negative values demonstrate a decrease in dopamine transporter function.

In both samples, associations were most pronounced between change scores in Ikelos‐RS and right AP (SBR and *z*‐score), with stronger correlations being observed after excluding confounding factors (Figure 3).

While change scores in CGI‐S did not show associations with any change score of DaT‐SPECT parameters in the total sample (‘all’), similar correlations to the Ikelos‐RS with change scores in the right AP (SBR: *r*_*s*_ = −0.379, *p* = 0.007; *z*‐score: *r*_*s*_ = −0.368, *p* = 0.008) and left PP (SBR: *r*_*s*_ = −0.293, *p* = 0.039; *z*‐score: *r*_*s*_ = −0.309, *p* = 0.029) were observed in the ‘cleaned’ sample.

## 4 Discussion

The present study evaluated key validation aspects of the Ikelos‐RS, determining the severity of RBD during the course of disease and treatment by expert interviewing of bed partners. Results for inter‐rater and test–retest reliability demonstrate high agreement between experts and reproducibility of ratings, confirming preliminary validation published in 2021 (Kunz et al. 2021). When evaluating construct validity, the Ikelos‐RS demonstrated high correlations with the CGI‐S. Videometric analysis showed a strong association between the Ikelos‐RS and RWA in PSG. Impressively, changes in Ikelos‐RS scores aligned with alterations in dopamine transporter density in the anterior and posterior putamen, with associations even more pronounced after adjusting for potential confounders.

Regarding its reliability, the Ikelos‐RS is comparable to the PSG‐based RBD Severity Scale (RBDSS; Sixel‐Döring et al. 2011). However, the Ikelos‐RS differs from the latter in its assessment of symptom expression, potentially allowing more accurate detection of severe cases by distinguishing complex movements with or without aggressive connotations and by integrating vocalisations into motor categories that are also weighted by aggressiveness.

Videometric analyses of the present study revealed that only 2.6% of complex motor activity during REM sleep was aggressive, which is consistent with previous studies (Frauscher et al. 2007, 2011). Other research also detected associations between motor aggressiveness and an increased hazard ratio for phenoconversion in iRBD (Nepozitek et al. 2021). These observations are in line with our results, showing that patients with aggressive complex behaviour during videometry (*n* = 10) had twice the RWA (mean = 36.9%) of patients without violent connotation (18.0%) and that 3 out of 4 patients with aggressive vocalisations (screaming) had noticeably higher RWA (range = 44.9%–82.7%) compared to the total sample (mean RWA = 27.5%).

Considering that different types of vocalisations in REM sleep are more likely to be distinguished by bedpartners in intensity than the various movements that can be performed even unseen under the blanket, evaluating vocalisation for aggressiveness might therefore provide more information in determining severity and prognosis of iRBD. This has already been acknowledged and implemented in other classification systems (Iranzo et al. 2005) and recommended in the guidelines of the international RBD Study Group (Cesari et al. 2022). The Ikelos‐RS categorises ‘leaving the bed’ as the most severe form of RBD behaviour. One may question why a simple ‘sliding out of bed’ scores higher severity than to beat, choke or bite bedpartners. From our experience in about 300 RBD patients over the past 10 years being treated with melatonin as a chronobiotic, we noticed a gradual effect that developed over weeks, sometimes months. The first RBD behaviour that stopped and rarely reoccurred was leaving the bed. Aggressive behaviour with injuries to the patient himself and bedpartner persisted more frequently during beginning of treatment, gradually decreasing over time. Moreover, ‘leaving the bed’ is a discrete event with 100% observational certainty, occurring in more advanced patients only. The assessment of ‘aggressiveness’ or ‘(risk of) injury’ is inherently subjective, depending on the individual sensitivities of both the patient and their bedpartner as well as experience of the interviewing expert. Our study restricted validation to patients with iRBD. Future work may focus on the use of Ikelos‐RS in patients with secondary RBD or even Non‐REM parasomnia.

Recently, the North American Prodromal Synucleinopathy (NAPS) consortium introduced a rating scale, the RBD Symptom Severity Scale (RBDSSS; Choudhury et al. 2024), which also relies on external anamnesis. The RBDSSS is self‐administered by bed partners, while the Ikelos‐RS is assessed by an expert through bed partner interview only. This difference may affect reporting and interpretation, while expert‐led assessment could enhance accuracy and consistency, minimising potential misinterpretations or incomplete reporting. Similar to our validation process, the construct validity of the RBDSSS was evaluated with the CGI‐S, revealing a demonstrably weaker correlation. While the REM sleep behaviour disorder questionnaire—Hong Kong (RBDQ‐HK; Li et al. 2010)—a self‐reported questionnaire for diagnostic and monitoring purposes—also demonstrates strong psychometric properties, the Ikelos‐RS offers a more comprehensive validation in comparison to both the RBDSSS and RBDQ‐HK, as it also exhibits associations with the gold‐standard biomarkers for α‐synucleinopathies in iRBD, RWA and DaT‐SPECT.

While the association between Ikelos‐RS and RWA was observed within videometric analyses for a small group and at baseline for the total sample, this was not confirmed in the group after controlling for confounders or in longitudinal analyses. Nevertheless, these findings support the RBD Study Group's proposed approach of incorporating videometry as a complement to RWA (Cesari et al. 2022) rather than replacing it.

Our data show that changes in dopamine transporter density in the putamen, but not in the caudate nucleus, appear to parallel alterations in the Ikelos‐RS measuring the severity of motor symptoms over time. This aligns with prior research suggesting the putamen has a stronger role in motor function (Arnaldi et al. 2024; Herz et al. 2021; Taniwaki et al. 2003), while the caudate nucleus appears more involved in cognitive processing (Müller et al. 2017; Provost et al. 2015).

Clinical symptoms of α‐synucleinopathies, like motor abnormalities or cognitive dysfunction, do not appear until 50%–80% of dopaminergic activity in the striatum is disrupted (Heng et al. 2023). In contrast, serial DaT‐SPECT imaging studies in healthy subjects and patients with iRBD or Parkinson's disease have shown a rather steady decline in DaT‐binding ratios in almost all individuals studied, which is accelerated in patients in more advanced prodromal states (Chahine et al. 2021; Iranzo et al. 2011; Simuni et al. 2018). Additionally, around 80% of iRBD patients develop clinical α‐synucleinopathies within a decade after diagnosis (Postuma et al. 2015), allowing a prolonged intervention period for using and evaluating treatments with the Ikelos‐RS.

Demonstrating that changes in Ikelos‐RS parallel trends in dopamine transporter density over time may indicate its usefulness for frequent clinical use and potential for assessing RBD progression. Its simple structure, with two scales assessing overall symptom severity and frequency, is designed for rapid completion also by a non‐RBD expert such as a general practitioner, making it a practical tool for routine clinical application. Future studies should investigate the utility of the Ikelos‐RS as a screening tool, potentially enabling earlier detection of RBD, especially considering that symptoms often precede diagnosis by years and could thus be identified during routine medical examinations rather than solely in specialised sleep centres.

In our prior reported observational study, courses of disease and treatment of 209 iRBD patients were rated using the Ikelos‐RS (Kunz et al. 2021), suggesting the potential of the Ikelos‐RS to capture changes in RBD symptoms over time. Notably, the study showed a gradual symptom improvement via Ikelos‐RS within four weeks with melatonin treatment according to a chronobiotic protocol. When confounding medication such as antidepressants or lipophilic beta‐blockers were co‐administered, improvement developed within 2 or 3 months. Additionally, symptoms remained stable after almost 5 years following discontinuation of medication, emphasising the Ikelos‐RS's relevance as a follow‐up parameter.

### 4.1 Limitations

RBD patients represent a cohort of advanced age (average age at diagnosis in this study was 68.40 ± 8.30 years), increasing the likelihood of bed partners being unavailable due to separation or death. In the present study, only 8/180 (4.4%) patients had no bed partners, making severity assessments partly reliant on alternative sources like visitors or home video recordings. Furthermore, strong associations between RWA and videometry‐derived Ikelos‐RS were found, but were less pronounced when correlated with the Ikelos‐RS from clinical practice in the total sample. This discrepancy may be attributed to a psychological effect, namely aggravation—the tendency to exaggerate symptoms. Patients often only seek medical attention after symptoms persist for several months or even years, causing distress to bed partners and seemingly exacerbated symptoms during initial consultations. This factor should be considered during patient history‐taking, alongside the potential influence of confounding factors such as psychotropic medication.

It should also be noted that the frequent initial use of the Ikelos‐RS may introduce recall bias, although this potential bias is likely to diminish over time due to the extended observation period (‘the last 6 months’).

Additionally, a limitation of this study is that the ordinalisation of the Ikelos‐RS score, which reflects the patient's retrospective recall of RBD frequency over an extended period, may not fully capture the variability in RBD frequency observed within a single night, as assessed by videometric analysis. To partially address this critical aspect, we controlled for RWA variability between the three PSG nights within one standard deviation for videometry.

Furthermore, exclusion of patients with PLMI ≥ 15 during videometric analysis may introduce selection bias, as PLMS is common in RBD. This criterion was chosen to enhance sample homogeneity and minimise confounding by excessive PLMS, potentially limiting generalisability to patients with higher PLMI values.

## 5 Conclusion

The present study confirms the reliability of the Ikelos‐RS and its association with the most relevant biomarkers of iRBD within testing multilevel construct validity. Using external anamnesis to provide information on the frequency and expression of RBD symptoms, the Ikelos‐RS may render an easy‐to‐use tool for evaluating RBD symptom severity in clinical settings and treatment effectiveness over time.

## Author Contributions

**Sophia Stotz:** writing – original draft, methodology, validation, formal analysis, conceptualization, visualization, software, investigation. **Frederik Bes:** supervision, writing – review and editing, project administration, visualization, conceptualization, investigation. **Dieter Kunz:** supervision, writing – review and editing, conceptualization, project administration, visualization, investigation.

## Ethics Statement

The ethics committee of Charité—Universitätsmedizin Berlin approved the publication of the results of the post hoc data analysis.

## Consent

Written informed consent was obtained from all patients.

## Conflicts of Interest

The authors declare no conflicts of interest.

## Data Availability

The data that support the findings of this study are available on request from the corresponding author. The data are not publicly available due to privacy or ethical restrictions.
